# Combining State-of-the-Art Pre-Trained Deep Learning Models: A Noble Approach for Skin Cancer Detection Using Max Voting Ensemble

**DOI:** 10.3390/diagnostics14010089

**Published:** 2023-12-30

**Authors:** Md. Mamun Hossain, Md. Moazzem Hossain, Most. Binoee Arefin, Fahima Akhtar, John Blake

**Affiliations:** 1 Department of Computer Science and Engineering, Bangladesh Army University of Science and Technology, Saidpur 5310, Bangladesh; 2 School of Computer Science and Engineering, University of Aizu, Aizuwakamatsu 965-8580, Japan

**Keywords:** skin cancer, medical imaging, classification, deep learning, transfer learning, max voting

## Abstract

Skin cancer poses a significant healthcare challenge, requiring precise and prompt diagnosis for effective treatment. While recent advances in deep learning have dramatically improved medical image analysis, including skin cancer classification, ensemble methods offer a pathway for further enhancing diagnostic accuracy. This study introduces a cutting-edge approach employing the Max Voting Ensemble Technique for robust skin cancer classification on ISIC 2018: Task 1-2 dataset. We incorporate a range of cutting-edge, pre-trained deep neural networks, including MobileNetV2, AlexNet, VGG16, ResNet50, DenseNet201, DenseNet121, InceptionV3, ResNet50V2, InceptionResNetV2, and Xception. These models have been extensively trained on skin cancer datasets, achieving individual accuracies ranging from 77.20% to 91.90%. Our method leverages the synergistic capabilities of these models by combining their complementary features to elevate classification performance further. In our approach, input images undergo preprocessing for model compatibility. The ensemble integrates the pre-trained models with their architectures and weights preserved. For each skin lesion image under examination, every model produces a prediction. These are subsequently aggregated using the max voting ensemble technique to yield the final classification, with the majority-voted class serving as the conclusive prediction. Through comprehensive testing on a diverse dataset, our ensemble outperformed individual models, attaining an accuracy of 93.18% and an AUC score of 0.9320, thus demonstrating superior diagnostic reliability and accuracy. We evaluated the effectiveness of our proposed method on the HAM10000 dataset to ensure its generalizability. Our ensemble method delivers a robust, reliable, and effective tool for the classification of skin cancer. By utilizing the power of advanced deep neural networks, we aim to assist healthcare professionals in achieving timely and accurate diagnoses, ultimately reducing mortality rates and enhancing patient outcomes.

## 1. Introduction

Skin cancer, a prevalent yet often misdiagnosed disease poses a significant challenge in medical diagnostics. Skin cancer is characterized by the uncontrolled growth of abnormal cells in the skin. The three most common types of skin cancer are basal cell carcinoma, squamous cell carcinoma, Merkel cell carcinoma, and melanoma [1]. Non-melanoma skin cancer is primarily caused by DNA damage from ultraviolet radiation exposure [2,3]. Skin cancer ranks among the most prevalent forms of cancer globally, making up around a third of all reported cancer diagnoses, with its frequency steadily rising each year [4]. Just in the United States, it is estimated that more than 9500 individuals receive a skin cancer diagnosis daily [5,6].

While skin cancer is often treatable, early detection and precise diagnosis play a pivotal role in achieving favourable treatment results and enhancing patient survival rates [7,8,9]. Skin cancer detection has traditionally relied on a mix of visual examination and histopathological analysis, methods which are fraught with limitations in accuracy and scalability. Unfortunately, traditional methods for diagnosing skin cancer, such as visual inspection and histopathological examination, can be time-consuming and subjective, and have high inter-observer variability.

Numerous non-invasive imaging technologies for skin cancer detection and monitoring have been developed [10] in recent years. One notable example is the use of multi-spectral sensors to detect differences in the refraction index using millimeter-wave to terahertzphotonic near-field imaging [11]. Recent advancements in different areas of artificial intelligence (AI) have significantly impacted many fields, suggesting that AI could greatly improve cancer diagnosis. The field of medical diagnostics is now benefiting from the rapid evolution of deep learning technologies, employing advanced models like Xception, InceptionResNetV2, and ResNet50V2 [12,13,14].

**Figure 1:**
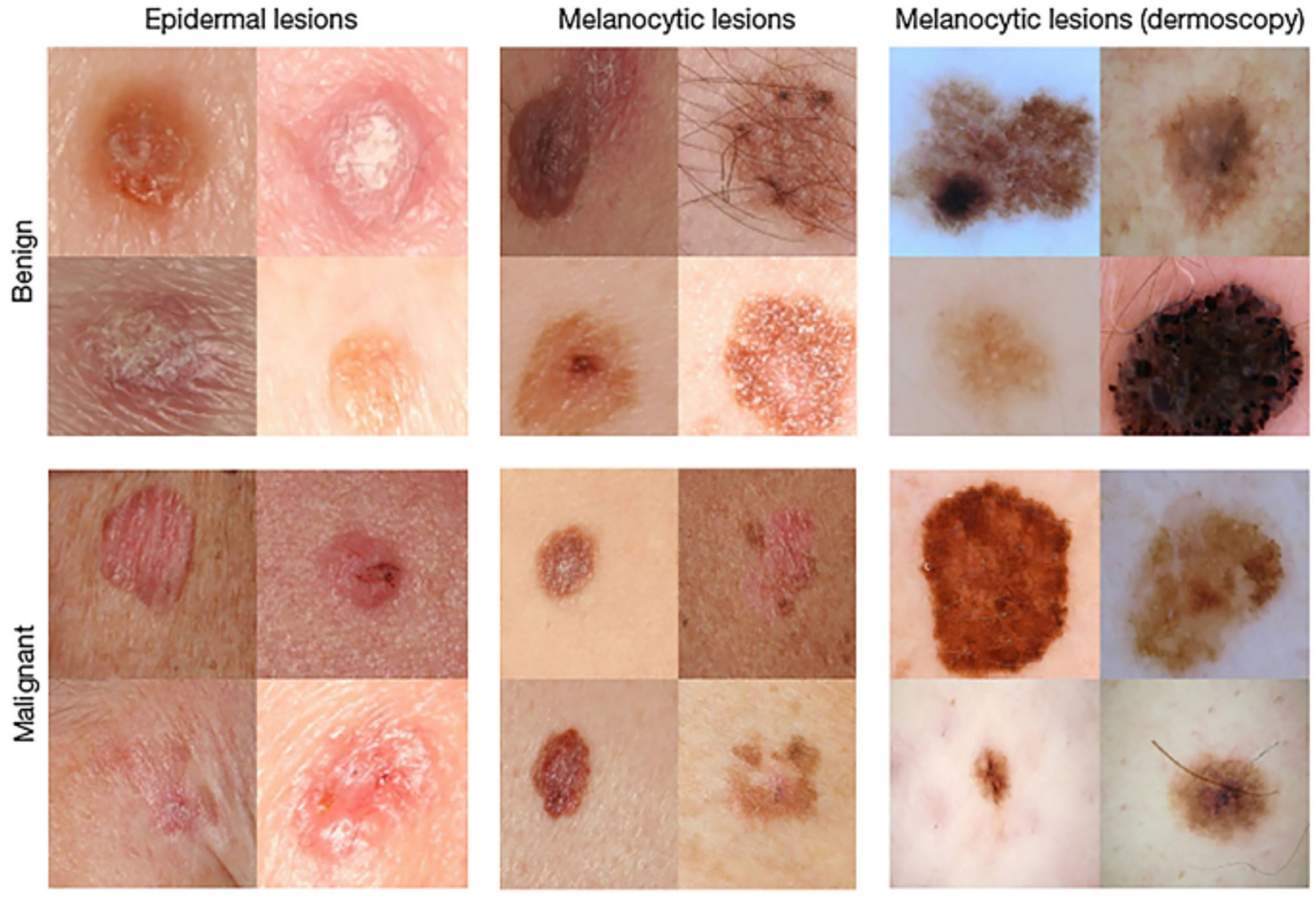
Skin Cancer—Malignant and benign sample images [3].

Despite these technological advancements, the difficulty in differentiating malignant from benign cases continues to hinder diagnostic accuracy. In recent years, deep neural networks, especially convolutional neural networks (CNNs), [15] have demonstrated significant potential in precisely detecting and categorizing skin cancer from medical images [7,16,17], such as those shown in Figure 1. CNNs are a specialized class of neural networks ideally suited for tasks involving image classification, as they can autonomously acquire hierarchical representations of image features directly from raw pixel values [18,19].

The challenge in skin cancer detection lies in the high variability of lesions and the limitations of current diagnostic methods, which often lead to inaccuracies and inconsistent diagnoses. Transfer learning, which entails the utilization of pre-trained CNN models for feature extraction from medical images, has additionally demonstrated enhancements in classification accuracy [20,21,22]. The application of deep learning methods in the domain of skin cancer detection and classification has emerged as a vibrant research area in recent years. Numerous investigations have substantiated that CNN models can achieve high accuracy in detecting and classifying skin cancer from medical images, and that transfer learning can further improve classification accuracy [23,24,25].

Although a substantial research gap still exists in the investigation of a holistic and cohesive methodology, the extant body of literature on skin cancer diagnosis utilizing deep learning models is rapidly growing with many researchers vying to create increasingly more accurate detection methods. There are, however, few studies that harness the possible synergies that may be achieved using a Max Voting Ensemble approach, which has not—as far as we know—been thoroughly investigated on a diverse dataset in the context of skin cancer diagnosis.

While deep learning for medical image analysis has made significant strides, a particular area of study interest is the application of cutting-edge pre-trained deep learning models to skin cancer detection. The primary aim of this work is to improve the precision and dependability of skin cancer diagnostic systems by examining the special use of the Max Voting Ensemble approach. This study presents an innovative approach for skin cancer detection and classification, utilizing the max voting ensemble technique with cutting-edge pre-trained deep learning models. The method proposed here combines the strengths of multiple pre-trained deep learning models, including Xception, InceptionResNetV2, ResNet50V2, InceptionV3, DenseNet121, DenseNet201, ResNet50, VGG16, AlexNet, and MobileNetV2, to create an ensemble with enhanced accuracy and robustness. The cutting-edge pre-trained models employed in this investigation have been extensively trained on large-scale skin cancer datasets, enabling them to capture complex features and patterns specific to skin lesions. By combining these models through the max voting ensemble technique, we aim to harness the diversity and complementarity of their predictive abilities, leading to improved generalization and discrimination power.

Our approach integrates multiple deep learning models, each selected for its proven strengths in image classification and feature extraction. Models such as Xception, ResNet50V2, and InceptionResNetV2 are renowned for their robustness in handling complex image data. This diverse selection ensures a comprehensive analysis of skin lesions, capturing a wide range of features from color and texture to shape and size variations. The synergy of these architectures enables a detailed analysis of dermatological images. The models underwent extensive training with augmented datasets, fine-tuning hyperparameters to balance bias and variance effectively, ensuring that the models are adept at identifying the subtleties of skin cancer lesions.

The max voting ensemble technique combines predictions from multiple deep learning models to derive a diagnosis. This technique harnesses the collective intelligence of the models. By aggregating the outputs, the ensemble approach effectively mitigates individual model biases and errors, leading to a more robust and reliable diagnostic tool. The Max Voting Ensemble is particularly adept at handling diverse and challenging skin lesion cases, offering adaptability and accuracy in various clinical scenarios. This adaptability is crucial in addressing the wide variability seen in skin cancer presentations. Empirical validation of our ensemble model underscores its superiority over individual models. The ensemble consistently demonstrated higher accuracy and diagnostic precision in our tests, which included a comprehensive comparison with the performance of standalone deep learning models.

Our research contributes to the ongoing evolution of artificial intelligence in medical diagnostics by addressing the demand for enhanced detection methods in dermatological health by examining the unexplored terrain of merging these advanced models. Our proposed noble approach holds the potential to transform the field of skin cancer identification and categorization, contributing to early diagnosis and more effective treatment strategies. With the integration of cutting-edge pre-trained deep learning models and the power of ensemble techniques, we aim to make a meaningful impact on healthcare and lead the way towards improved patient care and outcomes.

This research makes four significant contributions to the field of skin cancer detection and classification.

- Firstly, it successfully utilizes the max voting ensemble technique to effectively distinguish between benign and malignant skin cancer cases. This approach is notable for its ability to amalgamate the predictions of multiple models, thereby enhancing accuracy and demonstrating superior performance compared to individual models.
- Secondly, the study offers a comprehensive analysis of various deep learning models used for skin cancer classification. This analysis is rigorously assessed using the area under the receiver operating characteristic (ROC) curve (AUC), providing valuable insights into the effectiveness of these models in accurate disease identification.
- Furthermore, the ensemble method developed in this study not only surpasses the accuracy of single models but also showcases remarkable robustness and broad applicability across different datasets. This is evidenced by its ability to detect malignant cases with high precision in varied scenarios.
- In addition, the study introduces an innovative algorithm based on the max voting ensemble technique, integrating ten pre-trained individual models. These models collectively exhibit robustness and reliability, significantly outperforming most comparable alternatives. The efficacy of this ensemble approach is exemplified by achieving an AUC score of 0.932, underscoring its potential in medical diagnostics.

The results of this study can help guide the development of more accurate and efficient skin cancer detection and classification algorithms, which can ultimately lead to improved patient outcomes and survival rates. Beyond its immediate applications in skin cancer detection, our work carries broader implications for the field of medical image analysis. The successful integration of ensemble techniques could be extended to various medical imaging modalities and disease classifications. By leveraging the collective intelligence of multiple models, we can enhance the accuracy and reliability of diagnostic tools across diverse healthcare domains.

The organization of the remainder of this paper is as follows: the subsequent section presents an extensive review of the literature. Then, we introduce the materials and methods, describing the dataset, model architectures, and experimental setup. Subsequently, we present the results and analysis, showcasing the AUC scores of individual models and the Max Voting Ensemble. Finally, we discuss the ramifications of our findings, highlighting the significance of ensemble techniques in medical image analysis, particularly for skin cancer detection, and conclude with future research directions in this promising field.

## 2. Literature Review

Skin cancer ranks as the most prevalent form of cancer in the United States, accounting for approximately 5.4 million diagnosed cases each year. Skin cancer is often curable, but effective treatment is contingent on early detection. Delayed diagnosis and treatment can result in adverse outcomes, such as increased rates of metastasis, as well as heightened morbidity and mortality. Conventional approaches to skin cancer diagnosis, including visual inspection and biopsy, are characterized by subjectivity, time-intensive procedures, and the potential for significant inter-observer variation. Consequently, there has been a rising interest in leveraging machine learning methods, notably deep learning techniques, to enhance the precision and efficiency of skin cancer diagnosis. Over the past few years, deep learning methods [26], particularly convolutional neural networks (CNNs) [17,23,26,27,28], have demonstrated significant potential for accurately identifying and categorizing skin cancer from medical images of skin lesions [7,16,24,25]. In this review of relevant literature, we explore recent studies focusing on the detection and classification of skin cancer through the application of deep learning and transfer learning methodologies.

The advancements in deep learning techniques have spurred considerable progress in skin cancer detection. Recent review articles [29,30,31,32,33] explore key contributions in this domain, with a focus on the application of deep learning methods. For example, the comprehensive review by Dildar et al. (2021) [33] provides a contemporary overview of skin cancer detection leveraging deep learning techniques. The authors systematically analyze the latest advancements and methodologies in the field, offering valuable insights into the evolving landscape of skin cancer diagnostics. Brinker et al.’s [29] systematic review focuses on the application of convolutional neural networks (CNNs) for skin cancer classification. The paper critically assesses the state of the art in CNN-based skin cancer diagnosis, providing a comprehensive synthesis of existing literature and highlighting key trends in this rapidly evolving field. Adegun and Viriri (2021) conduct an in-depth exploration of deep learning techniques applied to skin lesion analysis and melanoma cancer detection [30]. Their survey not only summarizes existing methodologies but also critically examines the state of the art, offering insights into challenges and opportunities for further research in this domain. Munir et al.’s bibliographic review [31], featured in *Cancers*, presents a holistic examination of cancer diagnosis using deep learning, encompassing various cancer types, including skin cancer. The review consolidates knowledge from diverse studies, shedding light on the broad applications of deep learning in cancer diagnosis and underscoring its potential impact on improving diagnostic accuracy. Li et al. [32] conscientiously discuss the challenges faced in the application of deep learning to skin disease diagnosis. From data limitations to interpretability issues, the authors provide a balanced perspective on the obstacles that researchers and practitioners must navigate. Importantly, the review concludes with insights into potential future research directions, guiding the trajectory of advancements in this evolving field.

A number of recent studies of ensemble deep learning for biomedical imaging have advanced the field [34,35]. A case in point is Shokouhifar et al. [34], which used swarm intelligence empowered three-stage ensemble deep learning to measure arm volume as a difference in arm volume is an indicator of the presence of and change in the status of lymphedema. Another prime example is Bao et al. [35], who utilized integrated stack-ensemble deep learning to enhance the preoperative prediction of prostate cancer Gleason grade.

Numerous investigations have delved into the utilization of CNNs for the detection and categorization of skin cancer over the past two decades [7,32,35,36]. As an illustration, Esteva et al. (2017) trained a CNN model on a dataset comprising more than 129,000 clinical images to identify skin cancer. This model achieved a sensitivity of 95%, a specificity of 85%, and classification accuracy of 91% for melanoma detection, a performance level on par with that of dermatologists [23]. Likewise, Tschandl et al. (2018) employed a CNN model for the classification of skin lesions into benign or malignant categories, achieving an Area Under the Curve (AUC) score of 0.94 on an independent test dataset [37]. Brinker et al. (2019) conducted an assessment of various CNN models to detect melanoma, the most lethal type of skin cancer. Their findings highlighted a substantial enhancement in classification accuracy through the application of transfer learning with pre-trained CNN models [29].

Transfer learning, which encompasses the utilization of pre-trained CNN models to extract features from medical images, has also demonstrated an enhancement in classification accuracy when applied to the detection of skin cancer [23,24,25]. Han et al. (2018) developed a transfer learning-based CNN model that achieved a classification accuracy of 89.1% for melanoma detection using dermoscopic images [38]. Haenssle et al. (2018) used transfer learning to fine-tune a pre-trained VGG-19 model on a dataset of dermoscopic images to detect melanoma, achieving an AUC of 0.86 on an independent test set [38]. Codella et al. (2018) similarly used transfer learning to fine-tune a pre-trained Inception-v3 model on a dataset of dermoscopic images, achieving an AUC of 0.93 on an independent test set [39].

While numerous studies have showcased the potential of deep learning techniques for skin cancer detection and classification [29,30,31,32,33], it is necessary to compare the performance of various CNN models dedicated to this task. Comparative studies have focused specifically on comparing the performance of different CNN models for skin cancer detection and classification [7,9,14,40,41]. For example, Codella et al. (2018) compared the performance of three different CNN models (Inception-V3, ResNet50, and DenseNet-121) for classifying skin lesions as either benign or malignant. The authors found that DenseNet-121 achieved the highest classification accuracy, with an area under the receiver operating characteristic curve (AUC-ROC) of 0.91 [39,42].

Kawahara et al. (2018) used a pre-trained CNN model to extract features from skin lesion images and then trained a Support Vector Machine (SVM) classifier to distinguish between benign and malignant lesions. The authors found that their SVM classifier achieved an accuracy of 83.6%, which outperformed several other classification methods [43]. Brinker et al. (2019) compared the performance of five different CNN models on a dataset of dermoscopic images, finding that the Inception-v3 model achieved the highest AUC of 0.90 [29]. Patel et al.’s (2021) study suggests that transfer learning can improve the performance of CNN models for skin cancer detection and classification. Their findings also indicate that the InceptionV3 model may be particularly effective for this task [44]. Zhang et al. (2020) similarly compared the performance of six different CNN models on a dataset of dermoscopic images, finding that the DenseNet-121 model achieved the highest AUC of 0.95 [45]. Zaidan et al. (2021) reviewed the use of deep learning techniques for skin cancer detection and classification in their article published in the Journal of Healthcare Engineering. They discussed the effectiveness of different CNN models and transfer learning methods and the need for further research on larger and more diverse datasets [33].

Recent research endeavors have additionally investigated the application of alternative deep learning methodologies, including Generative Adversarial Networks (GANs), in the context of skin cancer detection and classification [46,47,48]. An illustrative example is Bi et al. (2020), who employed a GAN to generate synthetic skin lesion images and subsequently harnessed a CNN for their classification into benign or malignant categories. The researchers reported that their GAN-CNN model achieved a classification accuracy of 83% [49].

More recently, Guergueb and Akhloufi (2022) [50] used ensemble learning to achieve a predictive accuracy of just under 98% for melanoma disease, the most dangerous form of skin cancer. The authors used only one image dataset for training and testing and so the generalizability of their model to other datasets and other forms of skin cancer is untested. Avanija et al. (2023) [51] recorded an accuracy rate of 86% on the ISIC Skin Cancer Dataset using an ensemble learning approach harnessing three deep learning algorithms, namely, VGG16, CapsNet, and ResUNet. Even more recently, Sethanan et al. (2023) [8] report a cancer detection rate of 99.7% and cancer classification rates of approximately 96%. Their ensemble model harnesses modified CNN architectures, refined image segmentation techniques, and an artificial multiple intelligence system algorithm for optimized decision fusion. The authors, however, note that further research is needed to establish the generalizability, robustness, and clinical applicability of their model.

The max voting ensemble technique is a powerful approach within ensemble learning [8,34,35]. It aggregates predictions from multiple models and selects the class with the highest frequency of votes as the final prediction. This technique leverages the wisdom of crowds to arrive at a more accurate and stable prediction [52,53]. In the context of skin cancer detection, the max voting ensemble technique presents an opportunity to harness the collective intelligence of deep learning models and enhance classification outcomes [54]. For example, the study by Kausar et al. (2021) introduces deep learning-based ensemble models, achieving accuracies up to 91.8% using an individual-based model. However, using ensemble techniques boosted accuracy to 98% and 98.6%. The proposed models outperformed recent approaches, offering significant potential for enhanced multiclass skin cancer classification [55].

In summary, deep learning techniques, particularly CNNs and transfer learning, have shown great promise in accurately detecting and classifying skin cancer from medical images. Numerous studies have showcased the efficacy of CNN models for skin cancer detection and classification, and comparative studies have shown that certain CNN models, such as DenseNet-121 and Xception, achieve higher accuracy than others [56]. Transfer learning has also been shown to improve classification accuracy for skin cancer detection. However, further research is needed to evaluate the performance of different CNN models and transfer learning methods for skin cancer detection and classification on larger and more diverse datasets.

Table 1 below provides an overview of recent research conducted on diverse skin cancer datasets.

In Table 1, it is evident that researchers have conducted experiments utilizing a diverse assortment of pre-trained deep learning models across multiple skin cancer datasets, with a particular emphasis on ISIC 16, 17, 18, 19, 20, PH2, and HAM10000. These investigations have yielded a spectrum of accuracy scores. Notably, all datasets except ISIC 2018: Task 1-2 have demonstrated accuracy ranging from 95% to 99%. Conversely, ISIC 2018 falls short of this range with an accuracy below 92%. Consequently, our research focuses on the ISIC 2018: Task 1-2 dataset, aiming to enhance its accuracy to a level that meets acceptable standards.

## 3. Proposed Methodology

Skin cancer detection is a crucial task in medical diagnostics. This approach delineates an innovative method that harnesses cutting-edge pre-trained deep learning models within an ensemble framework, aimed at achieving precise skin cancer detection. The methodology entails the amalgamation of multiple pre-trained convolutional neural network (CNN) models through a maximum voting ensemble technique, thereby augmenting the overall classification performance.

### 3.1. Abstract View of Proposed Model

**Figure 2:**
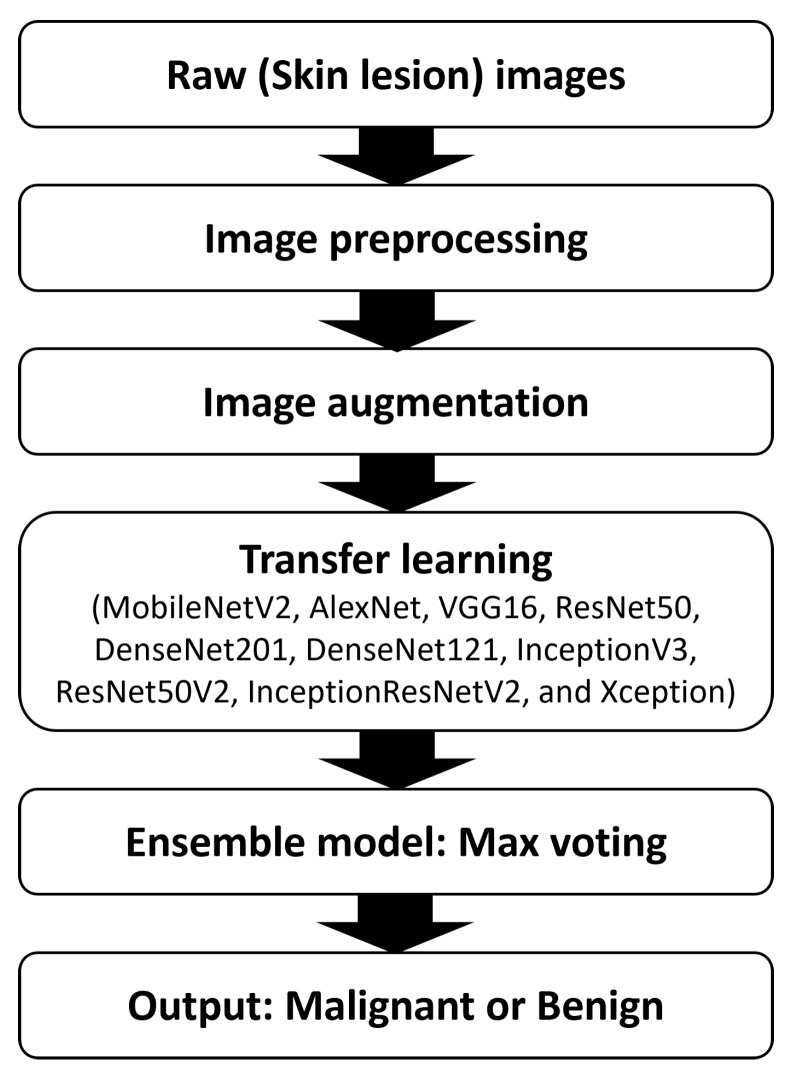
Abstract view of proposed Max Voting-based skin cancer classification.

The primary objective of this research is to investigate whether the proposed Max Voting-based ensemble model can more accurately distinguish between benign and malignant skin lesions than the individual models. Figure 2 shows the abstract view of the proposed Max Voting-based methodology using pre-trained models.

The essential stages of the method we propose are outlined below:

1. Dataset: Select a comprehensive skin cancer dataset containing a variety of benign and malignant skin lesion images. Common datasets including ISIC (International Skin Imaging Collaboration) are used to conduct the experiment.
2. Preprocessing: Ensure proper data preprocessing, including resizing, normalization, and data augmentation.
3. Data augmentation: During training, data augmentation techniques are employed to enhance learning outcomes and prevent overfitting.
4. Pre-trained Models: Choose a set of diverse pre-trained deep learning models for feature extraction. Models like MobileNetV2, ResNet50, InceptionV3, DenseNet201, and Xception are ideal due to their strong performance on image classification tasks. For each input image, extract features from intermediate layers of the selected pre-trained models. These features capture high-level representations of the image content.
5. Ensemble Construction: Implement a maximum voting ensemble technique to combine the predictions of individual models. For each test image, the ensemble generates predictions based on the majority vote from predictions made by the individual models.
6. Assess the ensemble’s performance on the test set using metrics including accuracy, precision, recall, F1-score, and ROC-AUC.

### 3.2. Detailed View of Proposed Model

The process of detection of benign and malignant lesions starts with image data collection, followed by steps such as image preprocessing, data augmentation, feature extraction through pre-trained models, and finally, classification by combining the outputs of individual pre-trained models through the proposed max voting-based ensemble model. Transfer learning was used in our models to reduce the computation time and encourage better results and performance.

**Figure 3:**
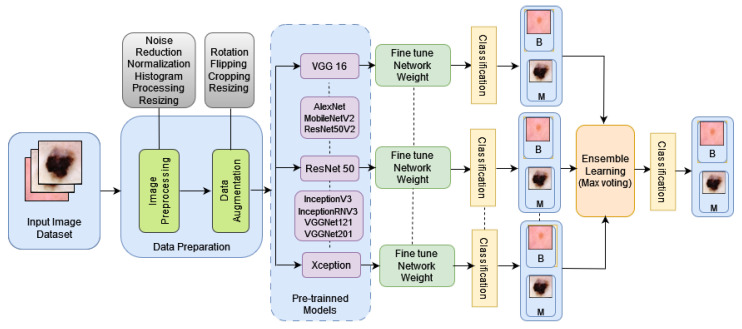
Overall workflow architecture of the proposed max voting-based ensemble technique.

This section outlines the detailed methodology we used to train and test our CNN models and predict the final outcome using the proposed max voting-based model as shown in Figure 3.

Figure 3 depicts the overall process of the proposed model for a skin lesion detection system. This process employs a max voting ensemble technique for classifying skin cancer using pre-trained deep-learning models. It begins by selecting a range of models, preprocessing input images, loading the chosen models, generating predictions for each model, aggregating predictions via majority voting, evaluating ensemble accuracy, and finally displaying the results. Algorithm 1 illustrates the data flow diagram of the model we propose, shown in Figure 3.
**Algorithm 1** Max Voting Ensemble Technique for Skin Cancer Classification**Input:**- List of pre-trained deep learning models (SelectedModels)List of skin lesion images (InputImages)Corresponding ground truth labels (groundTruthLabels)**Output**: Ensemble predictions for each input image (ensemble predictions)Step 1: Model SelectionSelectedModels←[MobileNetV2, AlexNet, VGG16, ResNet50, DenseNet201,DenseNet121, InceptionV3, ResNet50V2, InceptionResNetV2, Xception]Step 2: Preprocessing**while** InputImages≠eachImage **do**     PreprocessedImage←preprocessImage(image)     PreprocessedImages.append(PreprocessedImage)Step 3: Load Pre-trained ModelsLoadedModels←loadModels(SelectedModels)Step 4: Generate Predictions**while** eachpreprocessed≠PreprocessedImages **do**     predictions←[]     **while** eachloadedmodel≠LoadedModels **do**           ModelPrediction←PredictImage(LoadedModel,PreprocessedImage)           predictions.append(ModelPrediction)     individualPredictions.append(predictions)Step 5: Max Voting Ensemble Technique**while** eachIndividualPredictions≠individualPredictions **do**     aggregatedPredictions←[]     **while** eachImagePrediction≠setofpredictions **do**           majorityVote←calculateMajorityVote(imagePrediction)           aggregatedPredictions.append(majorityVote)     ensemblePredictions.append(aggregatedPredictions)Step 6: Evaluate Ensemble Performanceaccuracy←evaluateAccuracy(ensemblePredictions,groundTruthLabels)Step 7: Display Resultsprint(“EnsembleAccuracy:”,accuracy)

The max voting ensemble technique is a powerful and reliable method for skin cancer classification. The integration of pre-trained deep learning models aids in accurate and early diagnosis, contributes to improved patient outcomes and reduced mortality rates, and holds promising implications for medical image analysis and other diagnostic tasks.

This algorithm provides a conceptual overview of the approach outlined in the abstract. The implementation details, such as the specific code for loading models and conducting experiments, would require further elaboration based on the deep learning framework and tools being used.

### 3.3. ISIC 2018 Dataset: Task 1-2

**Figure 4:**
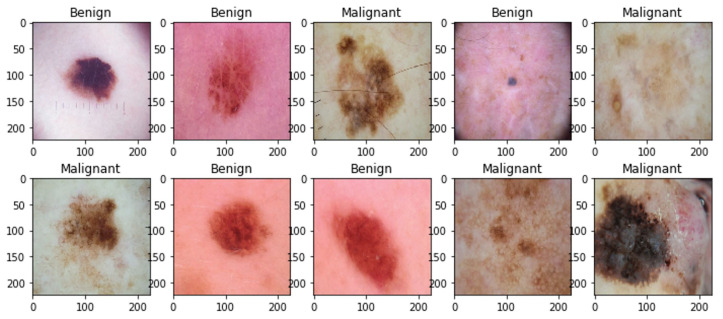
Skin Cancer—Malignant and benign sample images.

The ISIC 2018: Task 1-2 Skin Cancer Challenge Dataset publicly available at the ISIC Archive https://challenge.isic-archive.com/data/#2018 (accessed on 2 October 2023) [71] comprises an extensive compilation of dermoscopic images depicting skin lesions. These images are obtained from various sources and cover a range of skin conditions, including both benign and malignant cases. The dataset includes images with different lighting conditions, scales, and levels of image quality. The ISIC 2018 challenge is divided into three distinct tasks: Task 1 focuses on Lesion Segmentation, Task 2 involves Lesion Attribute Detection, and Task 3 addresses Disease Classification. The ISIC Challenge 2018: Task 1-2 datasets are systematically categorized into three segments, encompassing both benign and malignant cases. The Training dataset comprises 2597 images, with 2068 benign images, 519 malignant images, and 7 undetermined images. The Testing dataset includes 1000 images, featuring 794 benign images and 204 malignant images. The Validation dataset consists of 100 images. Figure 4 visually presents sample images depicting both malignant and benign cases from the ISIC 2018 Dataset of Task 1-2.

Here is a concise overview of the primary features of the ISIC 2018: Task 1-2 dataset:

- Annotations: Each image in the dataset is annotated with ground truth labels that indicate the type of skin lesion it depicts. These labels are typically categorized into classes such as melanoma, nevus, basal cell carcinoma, and others.
- Purpose: The dataset was released in conjunction with the ISIC Skin Cancer Detection Challenge in 2018. It was intended to serve as a benchmark for evaluating and comparing machine learning algorithms for skin cancer diagnosis.
- Usage: Researchers and participants in the challenge use this dataset to develop and train machine learning models, particularly deep learning models like convolutional neural networks (CNNs), for the accurate classification of skin lesions into different categories.
- Challenges: The ISIC community often organizes challenges based on these datasets to foster innovation in the field of skin cancer detection. Participants submit their algorithms for evaluation, and the results help advance the state of the art in automated skin lesion diagnosis.
- Availability: The dataset is made available to participants of the challenge and researchers interested in the field. It is often accessible through the ISIC website https://challenge.isic-archive.com/data/#2018 or challenge-specific platforms https://challenge.isic-archive.com/data/#2018 (accessed on 2 October 2023).

### 3.4. ISIC 2018 Dataset: Task 3 (HAM10000)

Discover the HAM10000 Skin Cancer Dataset [37]—a comprehensive collection of high-resolution images meticulously curated to aid in the early detection and diagnosis of various skin conditions. The HAM10000 dataset contains exactly 10,015 labelled images of seven skin disease classes: melanoma, melanocytic nevus, basal cell carcinoma, actinic keratosis, benign keratosis, dermatofibroma, and vascular lesion. It is possible to unleash the power of AI and dermatology with this invaluable resource, advancing skin cancer research and improving patient care worldwide. In this study, we performed experiments on the HAM10000 dataset to validate and extend the applicability of our proposed max voting-based ensemble technique to the ISIC 2018 dataset [71].

### 3.5. Image Preprocessing

Image preprocessing is the initial step where raw images are transformed to improve their quality and suitability for analysis. Preprocessing techniques for skin cancer classification include:

- Resizing and Cropping: Images are resized to a consistent resolution to ensure uniformity during model training.
- Rescale: Rescaling an image by dividing all pixel values by 255 is a common preprocessing step in image analysis and deep learning. The rescaling operation is particularly important when working with images represented in the RGB color space, where pixel values can range from 0 to 255 for each color channel.
- Normalization: Pixel values are normalized to a common range, such as [0, 1] through rescaling, to improve convergence during model training.
- Color Correction: Adjusting color balance and correcting lighting variations to reduce the impact of different acquisition conditions.
- Noise Reduction: Applying filters to reduce noise and enhance image clarity.

Table 2 summarizes the preprocessing required to conduct this research.

### 3.6. Data Augmentation

To enhance the performance and generalization capabilities of our image categorization model, we employed data augmentation techniques to artificially increase the diversity of training data by applying various transformations to the existing data. The goal is to improve the generalization and robustness of a machine learning model. This involved the strategic alteration of existing images in the training dataset. The augmentation process was carried out with careful consideration of several transformations, inspired by foundational works in the field [72,73,74,75]. The augmentation operations applied to the training dataset are summarized in Table 3. These operations were chosen based on their effectiveness in diversifying the dataset and improving the robustness of the model.

Our data augmentation strategy aligns with the findings and recommendations of key works in the literature. Mikołajczyk and Grochowski (2018) explored the benefits of data augmentation for improving deep learning in image classification problems, emphasizing the importance of exposing models to diverse data variations [72]. Perez and Wang (2017) further validated the effectiveness of data augmentation in image classification using deep learning through their comprehensive study [73]. In addition to these studies, the research report by Luis and Jason from Stanford University (2017) provided valuable insights into the practical effectiveness of data augmentation techniques in the context of image classification [74]. The survey by Shorten and Khoshgoftaar (2019) offered a broader perspective, summarizing various image data augmentation approaches and their impact on deep learning [75].

Our decision to employ data augmentation stems from its three-fold advantage. Firstly, it significantly expands the effective size of the training dataset, a particularly valuable asset when working with constrained data resources. Secondly, the exposure of the model to diverse variations in the training data enhances its ability to generalize to unseen scenarios. Thirdly, data augmentation acts as a regularization technique, mitigating overfitting risks by introducing controlled noise and diversity into the training data.

In conclusion, the augmentation strategy, informed by seminal works and a comprehensive survey, significantly contributes to the model’s performance, reliability, and generalization capabilities.

### 3.7. Usage of Pre-Trained CNN Models

A convolutional neural network (CNN) is a dedicated neural network architecture that integrates both convolutional layers and conventional neural network layers. Convolution, an operation that involves filtering images to extract features, is employed to enhance the model’s performance. Subsequently, a 3 × 3 MaxPooling filter is applied to obtain the most critical features. To enhance the model’s effectiveness, batch normalization and dropout layers are implemented to normalize the values and prevent overfitting.

The output from these layers is presented in a multi-dimensional matrix, which requires conversion to a one-dimensional form for input into the dense layer. This is accomplished using a flattened layer. The model was trained using the Rectified Linear Unit (ReLU) activation function within the initial dense layer. This choice was made to facilitate computation, as ReLU allows for the activation of only a few neurons at a time. In the second dense layer, the sigmoid function was employed, as it outputs a value between 0 and 1. This choice of activation function was made based on the specific requirements of the model and its intended use case. If the result exceeds 0.5, the classification is deemed malignant; otherwise, it is categorized as benign.

ImageNet was used, which we could use to load a pre-trained version of the network trained on more than a million images from the ImageNet database. In our preprocessing step, the batch size for our dataset was set to 32 and the image dimension to 224 × 224. A categorical class mode for our training data, which consisted of 2637 images, was used, while our testing dataset had 660 images. We then plotted our preprocessed images. Next, a pre-trained Model function from the Keras Applications class was imported and assigned to the variable ‘dense’. We specified “ImageNet” as the weights to be used and set the input shape to (224, 224, 3).

**Figure 5:**
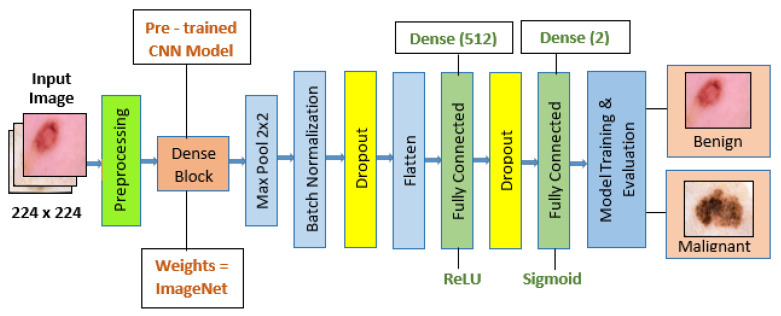
Flowchart depicting the steps involved in individual training and testing the models (Models: MobileNetV2, AlexNet, VGG16, ResNet50, DenseNet201, DenseNet121, InceptionV3, ResNet50V2, InceptionResNetV2, and Xception).

Figure 5 shows the flowchart depicting the steps involved in individual training and testing the model.

We formulated our model through a systematic layering approach within a sequential architecture, emphasizing the methodology without delving into the specifics of code functions. Firstly, we incorporated a pre-trained convolutional neural network (CNN) model, harnessing its feature extraction capabilities. This pre-trained model served as the foundation for our architecture. Subsequently, we applied max pooling with a 2 × 2 filter to facilitate spatial downsampling. This pooling operation allowed us to retain essential features while reducing computational complexity. By adhering to this structured methodology, we aimed to provide a comprehensive understanding of our model construction process, treating this document as an academic paper rather than a manual for code tools.

The subsequent subsection briefly outlines the pre-trained models employed in this study:

#### 3.7.1. AlexNet

AlexNet is a trailblazing deep convolutional neural network architecture renowned for its revolutionary advancements in image classification endeavors. It comprises a series of convolutional and pooling layers, culminating in fully connected layers. AlexNet distinguishes itself through the pioneering incorporation of ReLU activation functions and dropout regularization, contributing significantly to the prevention of overfitting. Although originally designed for general image classification, its principles have been adapted for skin cancer classification. By learning hierarchical features from skin images, AlexNet demonstrates effectiveness in distinguishing between different types of skin lesions, aiding in accurate diagnosis and classification [76].

#### 3.7.2. VGG16

VGG16 is a popular choice for tasks related to image recognition and classification. Its architecture comprises a total of 16 layers, encompassing 13 convolutional layers and 3 fully connected layers. Notably, all the convolutional layers employ a compact filter size of 3 × 3 with a stride of 1, facilitating precise feature localization within the input image. Additionally, the network incorporates max pooling layers with a 2 × 2 filter size to downsize the spatial dimensions of the output, effectively mitigating the risk of overfitting [77].

#### 3.7.3. InceptionV3

InceptionV3 is a convolutional neural network architecture renowned for its prowess in image classification tasks. In this context, InceptionV3 serves as a feature extractor, with the last layer substituted by a fully connected layer coupled with a sigmoid activation function, enabling the prediction of skin cancer probabilities. Training involves the utilization of a binary cross-entropy loss function, with optimization achieved through stochastic gradient descent [78].

#### 3.7.4. ResNet50

ResNet50 consists of 50 layers and is a variant of the ResNet architecture. It has a similar structure to other deep learning models. However, the unique feature of ResNet50 is its residual connection structure. The residual connections in ResNet50 help mitigate the problem of vanishing gradients by allowing the gradients to flow directly through the network. This enables the network to be trained deeper and more effectively, which has been shown to improve accuracy on image classification tasks [79].

#### 3.7.5. ResNet50V2

ResNet50V2, short for Residual Network 50 version 2, is a specific variant of the ResNet architecture that was introduced as an improvement over the original ResNet-50 model. The ResNet-50v2 architecture follows the same fundamental principles as the original ResNet, including the use of residual blocks and skip connections. However, it incorporates some modifications to the architecture and training process.ResNet-50v2 incorporates several enhancements aimed at improving training efficiency, convergence speed, and overall performance. Due to these enhancements, ResNet-50v2 generally achieves better accuracy and convergence speed compared to the original ResNet-50, especially on tasks like image classification [80].

#### 3.7.6. DenseNet121

DenseNet121 consists of 121 layers and is a variant of the DenseNet architecture. It has a similar structure to other deep learning models, with alternating convolutional and pooling layers, followed by a global average pooling layer and a fully connected layer for classification. However, the unique feature of DenseNet121 is its dense connectivity structure. The dense connectivity structure of DenseNet121 also enables it to efficiently learn complex features with fewer parameters compared to other deep learning models [81].

#### 3.7.7. DenseNet201

DenseNet201 is a powerful neural network architecture commonly used for skin cancer classification tasks. It is an extension of the DenseNet family, designed to enhance feature reuse and information flow across layers. With its densely connected blocks and efficient gradient propagation, DenseNet201 excels at capturing intricate patterns in skin images, aiding the accurate classification of various skin cancer types. Its deep structure and skip connections make it well suited for complex medical image analysis tasks like skin cancer detection [81].

#### 3.7.8. Xception

Xception shares similarities with InceptionV3 and has shown promising results for image classification tasks. One advantage of Xception is its ability to learn highly discriminative features with fewer parameters than other deep neural networks, which could lead to improved efficiency and reduced computational resources [82].

#### 3.7.9. MobileNetV2

MobileNetV2 stands out as a neural network architecture meticulously crafted for swift and efficient image classification, particularly on mobile and resource-constrained devices. It capitalizes on depthwise separable convolutions and linear bottlenecks to curtail computational complexity without compromising performance. MobileNetV2 strikes an optimal equilibrium between model size and accuracy, rendering it apt for real-time diagnosis and classification, even on devices with restricted resources. Its architectural design aligns with applications like skin cancer classification [83].

#### 3.7.10. InceptionResNetV2

InceptionResNetV2 is a sophisticated neural network architecture that combines elements from both Inception and ResNet designs. It leverages the advantages of both architectures, incorporating multi-scale feature extraction and residual connections. In skin cancer classification, InceptionResNetV2 excels at capturing intricate patterns within skin images and provides strong predictive capabilities. Its deep structure and complex modules make it well suited for detecting subtle characteristics indicative of various skin cancer types, enhancing classification accuracy and aiding in medical diagnoses [84,85]

The methodology employed in this study incorporates a diverse set of pre-trained deep learning models for skin cancer classification. Each model is selected based on its proven efficacy in image classification tasks and is fine-tuned to enhance its performance specifically for skin cancer diagnosis. The following fine-tuning and modifications are incorporated for the Skin Cancer Classification task:

1. Transfer Learning: The individual base model (e.g., Xception) is pre-trained on a large dataset for general image classification tasks, such as ImageNet. The knowledge gained from this pre-training is transferred to the skin cancer classification task.
2. Feature Extraction: Individual model (e.g., Xception) layer serves as a feature extractor, capturing hierarchical features from the input skin lesion images.
3. Additional Layers: Following the particular individual model (e.g., Xception) layer, additional layers such as convolutional, pooling, normalization, dropout, and dense layers are added. These layers contribute to further feature extraction, fine-tuning, and classification.
4. Dropout and Batch Normalization: Dropout layers are added to mitigate overfitting during training. Batch normalization is utilized for stable and accelerated training.
5. Output Layer: The final dense layer with two neurons (binary classification) is added for the skin cancer classification.

Table 4 shows the architectural summary of Xception (Functional) model along with parameters details as an example. The used Hyperparameters are—Learning Rate: 0.0001, Image Preprocessing Batch Size: 32, Varying Training Batch Sizes, Dropout Rates: 0.3 and 0.5, Optimizer: Adam, Loss Function: Categorical Cross-Entropy, callback Function: ReduceLROnPlateau with patience 1 and factor 0.5.

In summary, for each of these models, the process of adaptation generally involves:

- Pre-training: The models are pre-trained on a large dataset (e.g., ImageNet) to learn generic features.
- Fine-tuning: The pre-trained models are fine-tuned on a skin cancer dataset to adapt them to the specific task.

### 3.8. Max Voting Mechanism

Majority-based voting, often referred to as plurality voting, constitutes a prevalent approach within ensemble classification [86]. In this context, the EnsembleVoteClassifier technique stands out for its capacity to enhance overall performance and bolster prediction robustness through the amalgamation of multiple machine learning models—specifically, neural network classifiers. The mechanism hinges on a majority voting strategy, where each individual model in the ensemble contributes its prediction, ultimately culminating in a final prediction decided by the collective majority vote among these models.

The proposed approach herein revolves around the application of ten aforementioned pre-trained classification models, namely, MobileNetV2, AlexNet, seven instances of VGG16, ResNet50, DenseNet201, DenseNet121, InceptionV3, ResNet50V2, InceptionResNetV2, and Xception. To augment classification outcomes, this approach employs a majority-based voting mechanism. For each test instance, the classification results are independently computed by each of the specified models, and the ultimate output is predicted based on the outcomes that achieve majority representation [87,88,89,90]. In the context of majority voting, the class label y is forecasted by virtue of a majority (plurality) consensus reached by the individual classifiers, denoted as C.

$$\hat{y}=\mathrm{mode}\left\{C_{1}\left(x\right),C_{2}\left(x\right),\ldots ,C_{m}\left(x\right)\right\}$$

By following this comprehensive methodology, the proposed approach aims to provide a robust and accurate method for skin cancer detection using an ensemble of pre-trained deep learning models with a maximum voting scheme.

In summary, the max voting ensemble technique is chosen to address the limitations of individual models by combining their strengths, mitigating weaknesses, and providing a more robust and accurate solution for skin cancer detection. The ensemble approach capitalizes on the collective intelligence of multiple models to enhance diagnostic reliability and performance.

## 4. Experimental Evaluation

The subsequent subsection furnishes a comprehensive account of the experimental evaluation, elucidating the experimental setup, encompassing details such as hardware requirements, software environment, hyperparameters, and other relevant aspects.

### 4.1. Experimental Setup

The hardware environment utilized comprised Kaggle GPU resources, specifically T4x2, P100, and TPU: VM v3-8, each with a session duration of 12 hours, a disk capacity of 73 GB, RAM of 29 GB, and GPU memory of 16 GB. The software environments employed were Kaggle and Colab. The dataset utilized in this study was sourced from ISIC Challenge Datasets 2018: Task 1-2 [39,71], as obtaining skin lesion images from hospitals posed privacy and confidentiality concerns. For model training, 1440 benign and 1197 malignant skin lesion images from the ISIC Archive were employed. Model testing utilized 660 images, with 360 benign and 300 malignant images. Given the initial imbalance in the original image from the ISIC Archive https://challenge.isic-archive.com/data/#2018 (accessed on 2 October 2023), necessary adjustments were made to create a balanced set for both the training and testing sets. The original images were resized to 224 by 224, a measure taken to enhance model speed and performance. For ISIC 2018: Task 3 (HAM10000) Datasets, which are extremely imbalanced, we perform an oversampling operation to overcome the class imbalance problem and to ensure all the classes contain equal amount of images. Then, we employ Hold-Out Cross-Validation. The dataset is divided into training, validation, and test sets using an 80-10-10 split, with 80% of the data allocated to the training set, 10% to the test set, and 10% to the validation set.

Leveraging metrics such as training accuracy, training loss, validation accuracy, validation loss, true positive, true negative, false positive, false negative, precision, recall, and f1-score, we assessed the outcomes of our training process for the compiled model. The optimization was performed using the Adam optimizer with a learning rate set to 0.0001, and the loss function employed was categorical cross-entropy. We compiled the model with 11.6 million trainable and 85,000 non-trainable parameters for the DenseNet-121 model.

In our callback function, patience one and factor 0.5 for the ReduceLROnPlateau function were used. The model was then trained using both the train and validation datasets. As cross-validation was utilized, the test dataset was used as our validation dataset. For the DenseNet-121 and Xception models, we increased the training batch size to 64 and set the number of epochs to 20, which yielded improved accuracies. However, we did not specify any training batch size for other models and ran for 20 epochs.

Following the completion of training, the results of training and validation were gathered as seen in Table 5 and Table 6, respectively.Our model underwent evaluation using the test dataset and achieved accuracy consistent with the validation set. The results encompassed accuracy, precision, recall, F1-score, and loss metrics. Additionally, Mathews Correlation Coefficient, true positive, true negative, false positive, and false negative values were computed to assess the model’s capability. Accuracy, precision, recall, and F1-score are typically presented as percentages, providing a comprehensive evaluation of the model’s performance. On the other hand, the loss value is expressed as a scalar, representing the extent of the model’s error in predicting target values. Unlike other metrics, it is not constrained to a specific percentage range.

To evaluate the model’s predictions on the test dataset, we generated a list of predicted outputs. Subsequently, we visualized the accuracy and loss of our model through graphs. Additionally, a confusion matrix was generated to offer further insights into the model’s performance.

The hyperparameters used for the algorithm were a learning rate of 0.0001, an image preprocessing batch size of 32, and varying training batch sizes, with dropout rates of 0.3 and 0.5. The results obtained for all models are noted and compared in Table 5 and Table 6, respectively, and it was found that the highest accuracy was achieved using the Xception model. To further investigate the impact of different variables on the accuracy of the Xception model, several operations were performed and the resulting accuracy changes were recorded.

**Figure 6:**
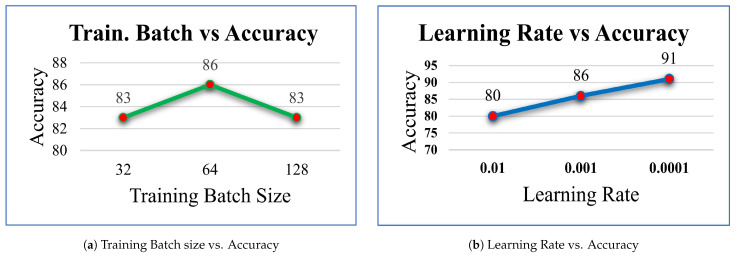
Training Batch size and Learning Rate versus Accuracy.

As part of our experimentation, we sought to determine how changes to the Training batch size affected the accuracy of our model. By systematically varying the batch size and recording the corresponding accuracy, it was found that increasing the batch size to 64 resulted in the highest accuracy, with a recorded value of 86%, as seen in Figure 6a.

For the next operation, the training batch size was fixed at 64 and the learning rate was varied to observe its effect on the model’s accuracy. Through this operation, we found that the highest accuracy of 91% was obtained when using a learning rate of 0.0001, as seen in Figure 6b.

**Figure 7:**
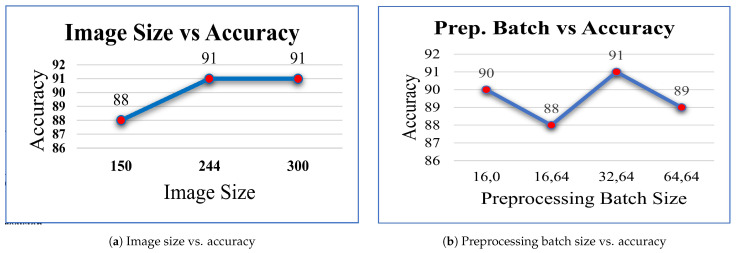
Image size vs. accuracy and preprocessing batch size vs. accuracy.

Subsequently, experiments were conducted to evaluate the impact of varying the image size on the model’s performance. Keeping the learning rate and training batch size fixed at 0.0001 and 64, respectively, the model was evaluated using different image sizes. It was found that for both 244 and 300 sizes, the model achieved similar accuracy results of 91% as seen in Figure 7a. However, as a smaller image size takes less time to process, we decided to keep the initial image size of 244 for our further experiments.

In order to optimize our model’s performance, we explored different combinations of inner and outer batch sizes while preprocessing the images. After testing various configurations, it was found that the initial combination of 32 image preprocessing and 64 training batch size resulted in the highest accuracy of 91% as seen in Figure 7b.

**Figure 8:**
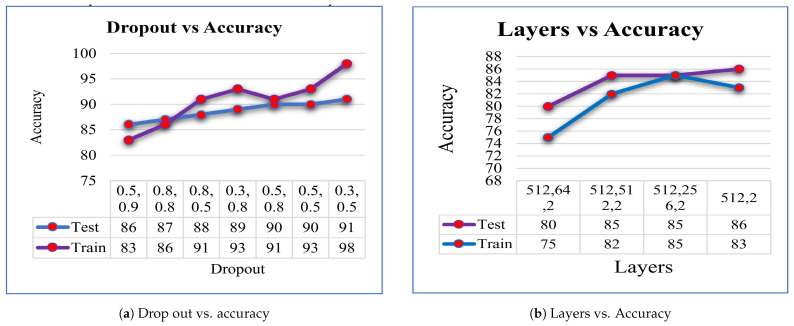
Dropout vs. accuracy and layers vs. accuracy.

To further improve the model’s performance and address the issue of overfitting, we experimented with different dropout rates as seen in Figure 8a. It was observed that using a lower rate for the first dropout and a higher rate for the second dropout helped overcome the overfitting problem. However, it was also found that increasing the dropout rate beyond a certain point led to a decrease in accuracy to some extent. These findings suggest that careful selection of dropout rates is crucial in achieving optimal model performance.

To improve the performance of our model, we added some more layers with dropout rates of 0.5 and 0.9, respectively, as seen in Figure 8b. Despite no overfitting issues, we did not observe any significant improvement in accuracy. In fact, increasing the number of layers resulted in a decrease in accuracy. There are several possible explanations for this, including diminishing returns, vanishing gradients, suboptimal hyperparameters, or limitations in the size or diversity of our dataset.

The aforementioned images correspond to the results obtained from running the Xception model for 30 epochs without specifying any training batch size. The identical model underwent several iterations with an unaltered dataset and a constant training batch size of 32 for 50, 100, 150, and 200 epochs, resulting nearly identical outputs throughout these runs.

### 4.2. Performance with ISIC 2018 Dataset: Task 1-2

In this study, following the application of various hyperparameter combinations, we evaluate the performance of ten distinct pre-trained deep learning models (MobileNetV2, AlexNet, VGG16, ResNet50, DenseNet201, DenseNet121, InceptionV3, ResNet50V2, InceptionResNetV2, and Xception). After fine-tuning all the hyperparameters, we assess the performance of the skin cancer classification method based on max voting through multiple evaluation metrics detailed in the subsequent subsections. We calculated the performance for both the training set and test set, which is noted in Table 7, and compared it with the proposed max voting ensemble technique.

The data aim to present the initial accuracies of various pre-trained CNN models on both the Training and Test sets. Each row in the dataset corresponds to a different model, and each column represents a specific dataset. The values in each cell of the table indicate the accuracy of the respective model on the corresponding dataset. For example, the accuracy of MobileNet V2 on the Train Set is 88.14%, and on the Test Set it is 77.20%. The accuracy of AlexNet on the Train Set is 89.40%, and on the Test Set it is 80.10%. Similarly, the percentages show the accuracy of each model on both the Train and Test sets.

The table is meant to provide a quick overview of how well each model performs on the given datasets, allowing us to compare their performance easily. This information could be used to evaluate and choose the most suitable model for the given task based on its performance on both the Train and Test sets. The data in the table highlight that the ensemble technique based on max voting yields superior accuracy compared to the individual models.

#### 4.2.1. Training and Validation Accuracy and Loss

**Figure 9:**
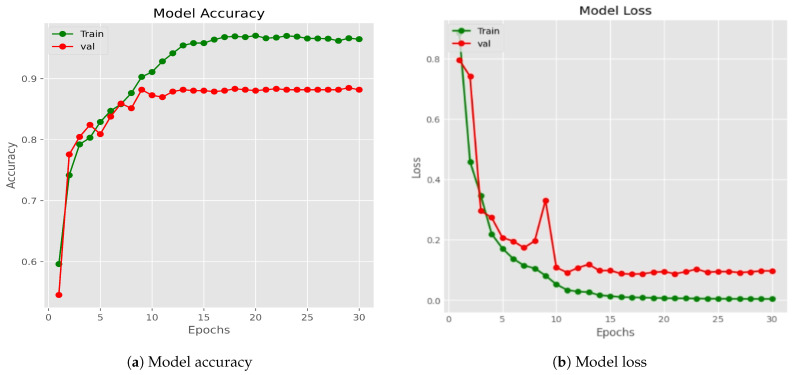
Accuracy and Loss Curve of Xception Model for 30 Epochs.

**Figure 10:**
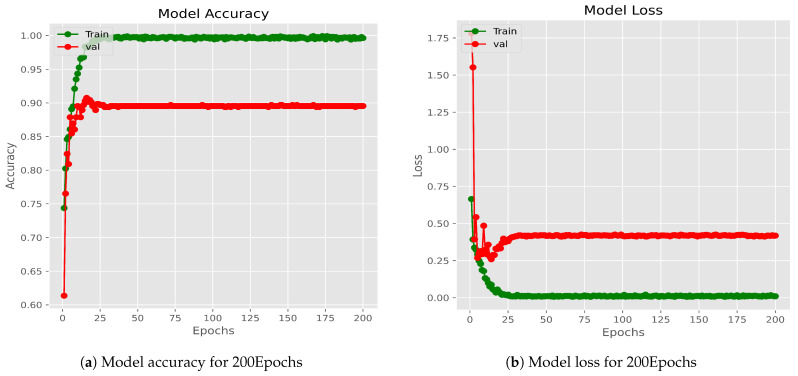
Accuracy and Loss Curve of Xception Model for 200 Epochs.

Figure 9 illustrates the progression of training and validation accuracy across epochs for the Xception model, which stands as the highest-performing individual model within the group of ten. From the outset, the training accuracy surpasses the validation accuracy, and this trend remains consistent up to epoch 30. Between epochs 1 and 20, both training and validation accuracy gradually ascend, with training accuracy reaching 100% and validation accuracy reaching 91%. Subsequently, the training accuracy experiences a sharp ascent from epoch 1 to 3, undergoes a substantial increase from epoch 3 to 20, and then stabilizes at 100% accuracy beyond the 20th epoch, depicted by the green line in Figure 10a. Similarly, the validation accuracy exhibits a sharp increase from epoch 1 to 3 but undergoes substantial fluctuation from epoch 3 to 20, and ultimately stabilizes beyond the 20th epoch, achieving a 91% accuracy level, represented by the red line in the same Figure. Between epochs 20 and 30, both training and validation accuracy maintain stability. Ultimately, the classifier model attains its highest accuracy at epoch 30.

In a similar vein, right from the start, the training loss consistently outperformed the validation loss, maintaining this pattern throughout all epochs. The training loss showed a swift decline from the initial epoch to the fourth, reaching a plateau at approximately 10 after the tenth epoch. In comparison, the validation loss experienced a rapid decrease from the first to the third epoch, followed by fluctuations between the fifth and tenth epochs, eventually settling at around 0.10, as depicted by the green and red lines in Figure 10b. Beyond the 30-epoch mark, the training loss converged to around 0.0037 while the validation loss stabilized at roughly 0.0943., with both values remaining constant.

The proposed system exhibited complete convergence by the 10th epoch, with the training loss hovering around 0 and the validation loss approximately at 0.10. These loss values remained nearly constant and exhibited a linear trend between epochs 10 and 30. The identical model underwent multiple executions utilizing the same dataset and a constant training batch size for 50, 100, 150, and 200 epochs, producing nearly identical outputs throughout these iterations. Figure 10 shows the accuracy and loss curve of Xception Model for 200 Epochs.

#### 4.2.2. Receiver Operator Characteristic (ROC)

**Figure 11:**
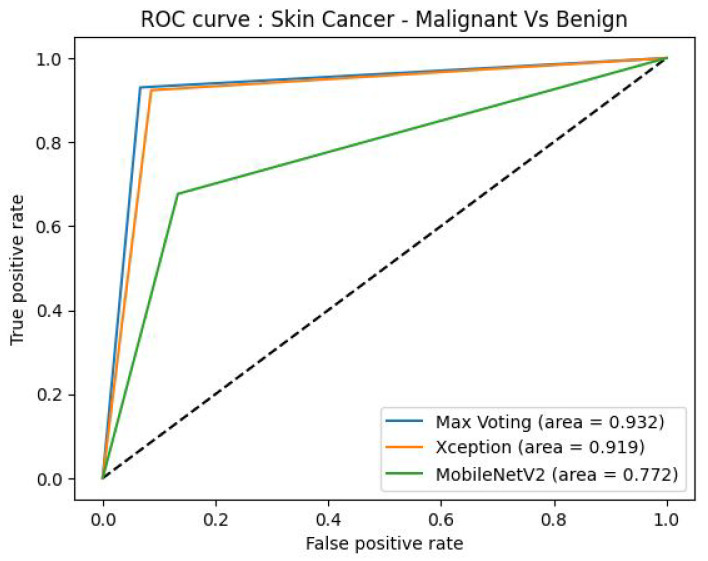
ROC Curve and AUC Scores for the MobileNetV2, Xception, and Max Voting.

The Receiver Operating Characteristic (ROC) is a graphical representation used to assess the performance of a classification model. In Figure 11, we depict the ROC curve for each category in our skin cancer dataset, along with the corresponding Area Under the Curve (AUC) values. On the graph, the x-axis represents the false positive rate, while the y-axis represents the true positive rate. The AUC values associated with each category provide insight into the probability values for those respective categories. The diagonal curve signifies random class probability selection. Among the individual models, the highest AUC value achieved is 91.90%, while the lowest is 77.00%, recorded by the Xception and MobileNetV2 models, respectively, as shown in Figure 11. These values are particularly relevant for two categories, namely malignant, and benign.

Figure 11 unmistakably demonstrates that the proposed ensemble model, based on majority voting, surpasses the performance of the individual models by achieving an AUC score of 93.20%.

#### 4.2.3. Error Analysis and Confusion Matrix (CM)

**Figure 12:**
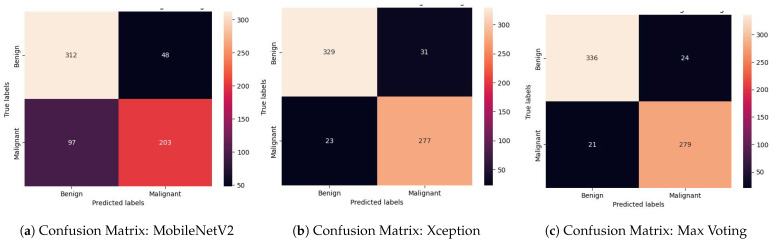
Confusion Matrix: Individual Models (MobileNetV2, Xception) Versus Proposed (Max Voting-based Ensemble) Model.

The confusion matrix (CM) serves as a tabular representation for summarizing a classifier’s performance [91]. In Figure 12, we present the confusion matrices for three distinct models: MobileNetV2 (Figure 12a), Xception (Figure 12b), and our proposed max voting-based ensemble model (Figure 12c). These matrices offer a consolidated view of prediction outcomes, distinguishing correct from incorrect classifications for two distinct classes, benign and malignant.

Within the matrix, counts are used to depict the number of accurate and erroneous predictions, categorized by class. The primary purpose of this matrix is to visualize classification errors and confusion that may arise during predictions. Specifically, the diagonal cells signify the count of accurate predictions aligning with the actual class, while the off-diagonal cells denote misclassifications into other classes. For instance, in Figure 12c, we assess the Benign class across a total of 360 unlabeled images. Among these, 336 images are correctly predicted as belonging to the Benign class, as evidenced by the first diagonal cell in Figure 12c. Conversely, 24 images are misclassified as malignant class. Similarly, we evaluate the Malignant class across 300 unlabeled images. Here, 279 images are correctly predicted as members of the Malignant class, shown in the fourth diagonal cell of Figure 12c, while 21 images are erroneously classified as benign classes.

#### 4.2.4. Comparative Analysis of Individual Models versus Proposed (Max Voting) Model

**Figure 13:**
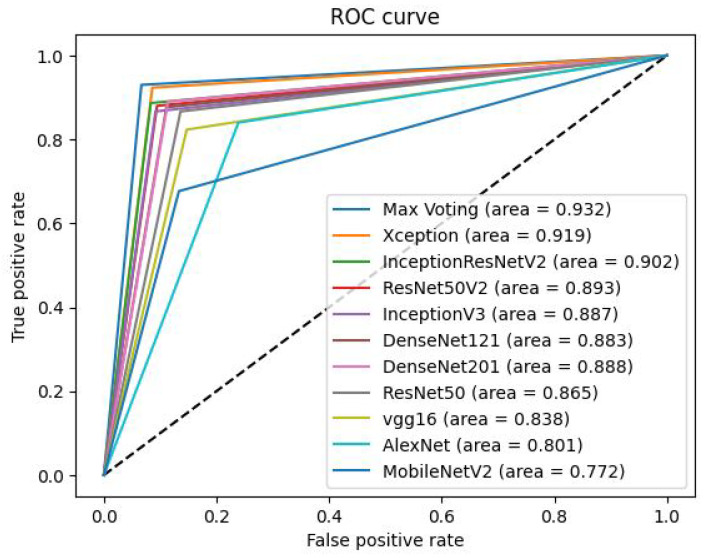
ROC Curve and AUC Scores for the models (Models: MobileNetV2, AlexNet, VGG16, ResNet50, DenseNet201, DenseNet201, InceptionV3, ResNet50V2, InceptionResNetV2, Xception and Max Voting).

The results of the skin cancer classification performance, measured by the area under the receiver operating characteristic (ROC) curve (AUC), for various deep learning models are depicted in Figure 13.

The performance of various deep learning models for skin cancer classification is quantified using the area under the receiver operating characteristic (ROC) curve (AUC) in which a higher AUC score indicates better classification performance. Table 8 provides a summary of the AUC scores for each model.

Three main similarities can be identified among these models, namely:

1. All models have AUC scores above 0.75, suggesting reasonably good classification abilities.
2. Architectural innovations, such as skip connections and dense connectivity, are common across most models.
3. Both ensemble and standalone models demonstrate strong performance.

Four differences may also be discerned. These are:

1. AUC scores range from 0.932 (Max Voting) to 0.772 (MobileNetV2), indicating different levels of effectiveness.
2. Computational complexity varies, affecting AUC scores.
3. Max voting, an ensemble technique, outperforms standalone models.
4. Newer architectures generally surpass older ones like AlexNet.

Our analysis reveals that while there are commonalities in high AUC scores and architectural innovations, the models also differ in computational complexity, intended use cases, and overall performance. Understanding these nuances can guide the choice of model for specific classification tasks in skin cancer detection.

The AUC metric is commonly used to evaluate the performance of binary classification models like skin cancer detection. A higher AUC indicates better discriminative power and better performance of the model in distinguishing between positive and negative samples. The provided AUC scores reveal the strengths and relative performance of different deep-learning models for skin cancer classification. Based on the AUC scores, the max voting ensemble achieves the highest performance with an AUC of 0.932, outperforming all individual models. The Xception model comes second with an AUC of 0.919, followed by InceptionResNetV2 with an AUC of 0.902.

These results indicate that the max voting ensemble technique, which combines predictions from multiple models, demonstrates superior performance in skin cancer classification compared to using individual models alone. The ensemble approach effectively leverages the strengths of different models, resulting in improved accuracy and generalization capabilities for skin cancer detection tasks.

Overall, the findings suggest that the max voting ensemble technique offers a promising approach to enhance the performance of skin cancer classification models using pre-trained deep learning models, potentially leading to better diagnostic capabilities in real-world applications. Researchers and practitioners can use these findings to select appropriate models for skin cancer classification tasks based on their specific requirements and resource constraints.

### 4.3. Classification Report Showing Accuracy, Precision, and Recall Score for the ISIC 2018 Datasets

The classification report, as depicted in Table 9, provides an overview of the performance of the proposed max voting ensemble model on the ISIC 2018 test datasets, encompassing a total of 660 images, including 360 benign and 300 malignant images, specifically highlighting the model’s performance in distinguishing between benign and malignant skin lesions. The report provides essential metrics such as precision, recall, and F1-score for each class, as well as overall accuracy, macro average, and weighted average values.

In the benign class (0), the model achieved a precision of 94.12%, indicating a high accuracy of positive predictions. The recall for benign instances stands at 93.33% and corresponding F1-score is reported at 93.72%. For the malignant class (1), the model achieved a precision of 92.08%, ensuring reliability in identifying malignant cases. The recall for malignant instances is 93.00% and the F1-score for the malignant class is 92.54%.

The overall accuracy of the model is reported at 93.18%, reflecting its correctness in classifying skin lesions. Macro average values of 93.10%, 93.17%, and 93.13% for precision, recall, and F1-score, respectively, provide an unbiased evaluation across both classes. The weighted averages, accounting for class imbalances are 93.19%, 93.18%, and 93.18% for precision, recall, and F1-score respectively.

In summary, the classification report offers a comprehensive assessment of the model’s effectiveness in classifying skin lesions in the ISIC 2018 datasets. The high precision, recall, and F1-score values for both benign and malignant classes, coupled with a notable accuracy of 93.18%, demonstrate the model’s robust performance in dermatological image analysis. The macro and weighted averages provide detailed insights on the distribution of instances across classes and offer a holistic view of the model’s capabilities in skin cancer classification. The reported accuracy of 93.18% indicates the overall correctness of the model’s predictions across classes.

### 4.4. State-of-the-Art Comparison for the ISIC 2018: Task 1-2 Datasets

The performance and accuracy of machine learning and deep learning models for medical image analysis, specifically in the context of detecting and classifying skin lesions, can vary depending on several factors, including the dataset used, the choice of model architecture, and the preprocessing techniques applied. The following subsection describes the performance of the proposed model with existing models. In recent years, few research activities have been carried out on skin cancer classification on the ISIC 2018: Task 1-2 dataset. The performance of the proposed system is compared with the previous approaches in terms of applied models and accuracy. Table 10 presents the testing accuracy values in existing approaches with our proposed method.

Table 10 indicates that the proposed approach achieves higher training and testing accuracy than the existing approaches of skin cancer classification using the ISIC 2018 dataset.

### 4.5. Performance with ISIC 2018: Task 3 (HAM10000) Dataset

The max voting ensemble technique introduced in this study for skin cancer classification exhibits significant potential for generalization to other related tasks within medical image analysis. Leveraging a comprehensive ensemble of pre-trained deep neural networks, including MobileNetV2, AlexNet, VGG16, ResNet50, DenseNet201, DenseNet121, InceptionV3, ResNet50V2, InceptionResNetV2, and Xception, this method showcases the adaptability of ensemble techniques for combining diverse model architectures. The success of the ensemble in elevating classification performance by aggregating predictions from individual models also suggests its applicability beyond skin cancer classification. With proper adaptation and training on relevant datasets, the max voting ensemble technique could be seamlessly extended to address diagnostic challenges in other medical domains, providing a versatile and effective tool for healthcare professionals in diverse diagnostic scenarios.

**Figure 14:**
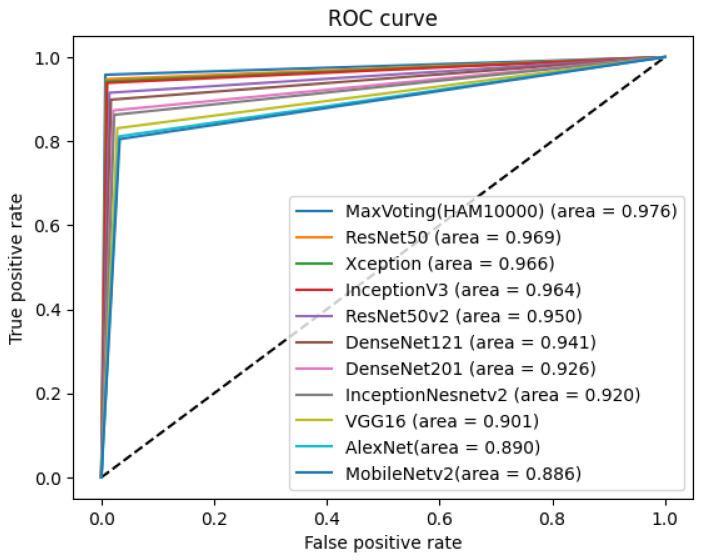
ROC Curve and AUC Scores for HAM10000 Dataset using proposed Max Voting Ensemble Technique.

Adapting the max voting ensemble technique for the HAM1000 dataset, which is a widely recognized dataset for skin image analysis, holds promise for enhancing the diagnostic capabilities of the ensemble method as shown in Figure 14. The ensemble’s incorporation of pre-trained deep neural networks, initially developed for skin cancer classification, aligns well with the diversity and complexity of the HAM1000 dataset, which comprises a large variety of skin lesions. Similar to the ISIC 2018: Task 1-2 dataset, the HAM10000 dataset attains improved accuracy through the amalgamation of individual models using the proposed technique.

**Figure 15:**
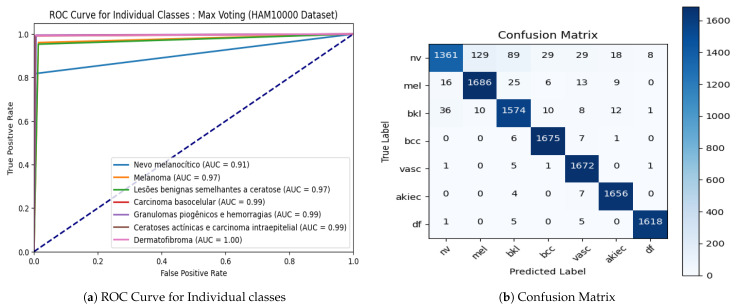
ROC Curve and Confusion Matrix using Max Voting Technique for HAM10000 Dataset.

Figure 15 shows the ROC curve with individual classes and confusion matrix for the max voting ensemble technique on the HAM10000 dataset. The proposed model is able to classify all seven skin decease classes, namely, melanoma, melanocytic nevus, basal cell carcinoma, actinic keratosis, benign keratosis, dermatofibroma, and vascular lesion with high accuracy, as shown in Figure 15a. The diagonal of the confusion matrix shown in Figure 15b indicates the high accuracy rate of the model as well.

### 4.6. Classification Report for the HAM10000 Datasets

Table 11 presents a detailed Classification Report for the HAM10000 datasets, summarizing the performance metrics for seven different classes (akiec, bcc, bkl, df, nv, vasc, and mel). For each class, the table includes precision, recall, F1-score, and support values, providing a comprehensive assessment of the model’s ability to classify skin lesions within each category. Additionally, the table reports the overall accuracy of the model, which stands at 95%, indicating the percentage of correctly predicted instances out of the total. Both macro and weighted averages are presented, offering unweighted and weighted evaluations across all classes, with each average showing consistent values of 0.95 for precision, recall, and F1-score. The support column reveals the distribution of instances among the different classes, totaling 11,734 instances in the dataset. This classification report offers valuable insights into the model’s performance on the HAM10000 datasets, crucial for understanding its effectiveness in dermatological image classification.

This approach demonstrates the potential for the broader application of ensemble methods in medical image analysis, contributing to the development of robust and reliable diagnostic tools across various healthcare domains.

## 5. Conclusions

In this study, we proposed a noble approach of employing the max voting ensemble technique with cutting-edge pre-trained deep learning models for skin cancer detection and classification. This study has made significant contributions to the field of skin cancer detection and medical image analysis. Our primary focus has been on enhancing both the accuracy and reliability of skin cancer diagnosis and we have achieved this through the innovative application of the max voting ensemble technique in conjunction with state-of-the-art pre-trained deep learning models. The main contribution of our research lies in the successful utilization of the max voting ensemble technique. By combining the predictions of multiple models, we have demonstrated its potential to significantly elevate the diagnostic performance in the context of skin cancer detection. Through extensive experimentation and rigorous analysis, we have shown that our ensemble method outperforms individual models. With an AUC of 0.932, it has established itself as a powerful tool for improving the accuracy and reliability of skin cancer classification. Our study offers insights into the selection of cutting-edge pre-trained deep learning models for single lesions. Models such as Xception, InceptionResNetV2, and ResNet50V2 have proven to be highly effective in capturing intricate features and patterns relevant to skin cancer detection. Beyond its immediate applications in skin cancer detection, our work carries broader implications for the field of medical image analysis. The successful integration of ensemble techniques can be extended to various medical imaging modalities and disease classifications. By leveraging the collective intelligence of multiple models, the accuracy and reliability of diagnostic tools across diverse healthcare domains can be enhanced. Moreover, the max voting ensemble technique serves as a testament to the potential of ensemble learning in medical image analysis. As we continue to advance in this field, ensemble techniques are likely to play an increasingly vital role in improving the quality of healthcare, aiding healthcare professionals in making more accurate and timely diagnoses, and ultimately contributing to better patient outcomes. Our research not only addresses the critical challenge of skin cancer detection but also provides a framework for the integration of ensemble techniques in medical image analysis.

The study primarily focuses on skin cancer detection using the ISIC 2018: Task 1-2 dataset. While this dataset is widely used and provides valuable insights, the generalizability of the proposed max voting ensemble technique may be influenced by several factors. These include the characteristics of the dataset such as image quality, class imbalance, and algorithmic diversity, as well as evaluation metrics and computational resources. Additionally, potential biases in the dataset, particularly the underrepresentation of certain skin types, ages, or ethnic groups, could limit the accuracy of the model across diverse populations. Although we assess the validity and generalizability of the proposed model with the HAM10000 dataset, further research could explore the effectiveness of the technique on diverse datasets representing different demographics and skin conditions. Furthermore, the challenges in ensuring algorithmic transparency and interpretability are crucial, especially in healthcare applications, for gaining trust from professionals and patients. The study mainly evaluates the proposed approach using quantitative metrics. To establish its real-world clinical utility, further validation through collaboration with healthcare professionals and clinical trials is necessary. This should include continuous evaluation of the performance of the model over time and its adaptability to evolving medical guidelines and emerging skin conditions. Moreover, regulatory challenges and ethical considerations, such as obtaining approval and addressing patient consent and data privacy, are essential factors to consider. The resource requirements for implementing and maintaining such a system in clinical settings, especially in resource-limited environments, also present significant limitations. The integration of AI tools into clinical workflows might face challenges, including the reception by healthcare professionals and the need for training and adaptation to new technologies. Lastly, a comparison with existing diagnostic methods in terms of accuracy, efficiency, and cost is imperative to understand the potential limitations in outperforming or integrating with current diagnostic processes.

Our research focused on the categorization of images of single lesions as either benign or cancerous. However, it should be noted that other approaches place the focus on wide-field or total-body imaging. Soenksen et al. (2021) cogently argue that a whole body approach may better replicate the practise of medical practitioners, who take into account a multitude of factors, such as the context, number and type of lesions when identifying lesions as suspicious [92]. Several studies using total-body imaging have shown fruitful results. Betz et al. (2022) on a study of 10 patients and analysis of slightly under 5000 lesions achieved 70% agreement with the gold standard. Their focus, however, was on counting the number of naevi [93] among the lesions. Strzelecki et al. (2021) studied the effectiveness of three algorithms developed to detect and segment lesions using whole-body imaging, and report promising results particularly for lesions greater than 3 mm [94]. Birkenfeld et al. (2020) used whole-body visual examinations to classify pigmented lesions as suspicious or non-suspicious, and propose that this approach may be adopted as a preliminary method to screen a population for further testing [95]. A direct comparison between total-body-imaging approaches and single-lesion approaches is difficult, given the difference in tasks. Single-lesion approaches tend to adopt dichotomous classification tasks of either benign or cancerous, while total-body imaging studies do not tend to assign such values to individual lesions, but adopt different tasks, such as calculating the number of naevi [93], and comparing the performance of different algorithms [94]. A single-lesion approach is highly effective in offering a detailed and focused analysis of individual lesions, which is crucial for precise diagnosis of specific skin conditions. However, its limitation lies in the potential oversight of the broader context of skin health, as it fails to consider the overall distribution, number, or variety of lesions across the body, which can be critical in identifying patterns indicative of systemic health issues. On the other hand, the total-body imaging approach stands out for its ability to provide a comprehensive overview of the skin, facilitating the detection of patterns and multiple lesions that are essential in diagnosing conditions spread over larger skin areas. This method, however, may compromise on the level of detail for individual lesions, potentially affecting the accuracy in diagnosing certain types of skin anomalies that require further examination.

In future work, we aim to seek out ways to address these limitations and to explore more sophisticated algorithms. Our next step will be to test the efficacy of including the integration of a weighted averaging technique, and assess its impact on classification performance with respect to the current state-of-the-art models. Another key focus will be on diversifying the datasets used in our models to include a broader range of skin types, ages, and ethnic backgrounds, thereby reducing bias and improving the generalizability of our findings. We plan to continue advancing the field of medical image analysis and enhancing healthcare outcomes. In addition to addressing the pressing challenge of skin cancer detection, our work serves as a catalyst for future innovation, ultimately contributing to the improvement of healthcare delivery and the preservation of lives through enhanced diagnostic accuracy, and achieving the ultimate goal of enhancing healthcare delivery and saving lives through improved diagnostic accuracy.

## Figures and Tables

**Table 1:** Recently conducted research on various skin cancer datasets.

Authors and Paper	Dataset	Model	Published Year	Performance
Gajera et al. [57]	ISIC 2016, 2017, PH2, HAM10000	AlexNet, VGG16, VGG19	2023	Accuracy = 98.33%, F1 score = 96%
Alenezi et al. [58]	ISIC 2017, HAM10000	deep residual network	2023	Accuracy = 96.97%
Inthiyaz et al. [59]	Xiangya-Derm	CNN	2023	AUC = 0.87
Alwakid et al. [60]	HAM10000	CNN, ResNet50	2023	F1-score = 0.859 (CNN), 0.852 (ResNet50)
Alenezi et al. [61]	ISIC 2019, 2020	ResNet-101 and SVM	2023	Accuracy = 96.15% (ISIC19), 97.15% (ISIC20)%
Abbas and Gul [62]	ISIC 2020	NASNet	2022	Accuracy = 97.7%, F1-score = 0.97%
Abdar et al. [12]	ISIC 2019	ResNet15V2, MobileNetV2	2021	Accuracy = 89%, F1-score = 0.91
Jain et al. [13]	HAM10000	Xception, InceptionV3, VGG19, ResNet50, and MobileNet	2021	Accuracy = 90.48% (Xception)
Aljohani and Turki [14]	ISIC 2019	Xception, DenseNet201, ResNet50V2, MobileNetV2, VGG16, VGG19, and GoogleNet	2022	Accuracy = 76.09%
Bechelli and Delhommelle [63]	HAM10000	CNN, VGG16, Xception, ResNet50	2022	Accuracy = 88% (VGG16)
Demir et al. [64]	ISIC archive	ResNet101 and InceptionV3	2019	F1-score = 84.09% (ResNet101) and 87.42% (InceptionV3)
Rashid et al. [65]	ISIC 2020	MobileNetV2	2022	accuracy = 98.20%
Reis et al. [66]	HAM10000, ISIC 2019, 2020	InSiNet, U-Net	2022	Accuracy= 94.59% (HAM10000), 91.89% (ISIC2019), and 90.54% (ISIC2020)
Khan et al. [67]	ISBI 16, 17, 18, PH2, HAM10000	ResNet101, DenseNet201	2021	Accuracy = 98.70% (PH2), Accuracy = 98.70% (HAM10000)
Khan et al. [68]	ISBI 2018,	A hybrid model	2021	Accuracy = 92.70%
Kaggle Compt. [69]	ISIC 2018	Top 10 model Average	2020	Accuracy =86.7%
Gouda et al. [70]	ISIC 2018	CNN	2022	Accuracy = 83.2%

**Table 2:** Preprocessing operations for test dataset.

Processing	Mode (Range)
Resizing	224 × 224
Rescale	1.0/255
Normalize	[0, 1]

**Table 3:** Augmentation operations for training set.

Processing	Mode (Range)
Rescale	1.0/255
Rotation range	0.2
Width shift range	0.2
Shear range	0.3
Zoom range	0.3
Fill mode	Nearest
Horizontal flip	True

**Table 4:** Architecture of Xception (Functional) model.

Layer (Type)	Output Shape	Number of Parameter(s)
xception (Functional)	(None, 7, 7, 2048)	20,861,480
conv2d_14 (Conv2D)	(None, 5, 5, 32)	589,856
max_pooling2d_2 (MaxPooling 2D)	(None, 2, 2, 32)	0
batch_normalization_14 (BatchNormalization)	(None, 2, 2, 32)	128
dropout_6 (Dropout)	(None, 2, 2, 32)	0
flatten_2 (Flatten)	(None, 128)	0
dense_6 (Dense)	(None, 512)	66,048
dropout_7 (Dropout)	(None, 512)	0
dense_7 (Dense)	(None, 256)	131,328
dropout_8 (Dropout)	(None, 256)	0
dense_8 (Dense)	(None, 2)	514
Total Parameter:		21,649,354
Trainable Parameter:		21,594,762
Non-trainable Parameter:		54,592

**Table 5:** Performance of the training dataset.

Metrics	MobileNet V2	AlexNet	vgg16	ResNet50	DenseNet 121	DenseNet 201	InceptionV3	ResNet 50V2	Inception ResNetV2	Xception
Accuracy	88.14%	89.40%	93.70%	94.50%	95.10%	95.80%	95.37%	96.30%	97.20%	99.20%
Precision	87.74%	89.10%	93.20%	93.65%	94.55%	95.20%	94.44%	96.00%	96.44%	99.20%
Recall	88.44%	90.14%	94.17%	95.30%	95.90%	96.00%	96.02%	96.95%	98.10%	99.40%
F1	88.94%	89.20%	93.60%	94.30%	95.05%	95.70%	95.34%	96.21%	96.70%	99.20%
Loss	0.1715	0.1646	0.1555	0.1423	0.1306	0.1237	0.1142	0.0820	0.0703	0.0037

**Table 6:** Performance of the testing dataset.

Metrics	MobileNet V2	AlexNet	vgg16	ResNet50	DenseNet 121	DenseNet 201	InceptionV3	ResNet 50V2	Inception ResNetV2	Xception
Accuracy	77.20%	80.10%	83.80%	86.05%	88.30%	88.80%	89.50%	89.30%	90.20%	91.90%
Precision	77.00%	79.80%	83.60%	85.65%	88.10%	88.20%	88.94%	89.10%	89.80%	91.20%
Recall	78.10%	81.00%	84.20%	86.95%	88.80%	89.20%	90.15%	90.10%	91.10%	92.10%
F1	77.10%	80.00%	83.70%	86.01%	88.10%	88.50%	89.47%	89.20%	90.10%	91.80%
Loss	0.3310	0.3156	0.3045	0.3023	0.2706	0.2657	0.2481	0.2427	0.1213	0.0943

**Table 7:** Initial accuracies of Train and Test set.

Set	Mobile- NetV2	AlexNet	vgg16	ResNet50	DenseNet 201	DenseNet 201	Inception V3	ResNet 50V2	Inception ResNetV2	Xception	Max Voting
Train	88.14%	89.40%	93.70%	94.50%	95.10%	95.80%	95.70%	96.30%	97.20%	99.90%	-
Test	77.20%	80.10%	83.80%	86.50%	88.30%	88.80%	88.70%	89.30%	90.20%	91.90%	93.18%

**Table 8:** Summary of AUC Scores and Key Features of Deep Learning Models for ISIC 2018 dataset.

Model	AUC Score	Key Features
Max Voting	0.932	Ensemble Technique
Xception	0.919	Modified Inception Architecture
InceptionResNetV2	0.902	Inception + ResNet
ResNet50V2	0.893	Improved ResNet with Skip Connections
InceptionV3	0.887	Multi-level Feature Extraction
DenseNet121	0.883	Dense Connectivity
DenseNet201	0.888	Dense Connectivity
ResNet50	0.865	Skip Connections
VGG16	0.838	Classic Deep Learning Model
AlexNet	0.801	Pioneering Deep Learning Model
MobileNetV2	0.772	Lightweight Architecture

**Table 9:** Classification Report for the ISIC 2018 datasets.

	Precision	Recall	F1-Score	Support
Benign	94.12	93.33	93.72	360
Malignant	92.08	93.00	92.54	300
Accuracy			93.18	660
Macro avg	93.10	93.17	93.13	660
Weighted avg	93.19	93.18	93.18	660

**Table 10:** Comparative analysis of ISIC 2018: Task 1-2 dataset used for skin cancer classification.

Authors and Paper	Year	Model	Accuracy
Gouda et al. [70]	2021	CNN	83.2%
Kaggle Compt. (3rd Place) [69]	2020	Individual Single Model	84.5%
Kaggle Compt. (2nd Place) [69]	2020	Meta Ensemble	86.3%
Kaggle Compt. (1st Place) [69]	2020	Top 10 model Average	86.7%
Khan et al. [68]	2022	A hybrid CNN Model	92.70%
Proposed method	2023	Max Voting ensemble model	93.18%

**Table 11:** Classification Report for the HAM10000 dataset.

	Precision	Recall	F1-Score	Support
class 0: akiec	0.98	0.98	0.98	1667
class 1: bcc	0.96	0.98	0.97	1689
class 2: bkl	0.94	0.94	0.94	1651
class 3: df	0.99	0.98	0.99	1629
class 4: nv	0.89	0.82	0.85	1663
class 5: vasc	0.94	0.98	0.96	1680
class 6: mel	0.92	0.95	0.94	1755
Accuracy			0.95	11,734
Macro Avg.	0.95	0.95	0.95	11,734
Weighted Avg.	0.95	0.95	0.95	11,734

## Data Availability

The data presented in this study are openly available at the ISIC Challenge Datasets website: https://challenge.isic-archive.com/data/#2018 (accessed on 2 October 2023) and, the ISIC Archive Datasets websites: https://api.isic-archive.com/collections/63/ and https://api.isic-archive.com/collections/212/ (accessed on 2 October 2023).

## Abbreviations

The following abbreviations are used in this manuscript:

AlexNet	Alex Krizhevsky’s Network
AUC	Area Under the Curve
CNN	Convolutional Neural Networks
DenseNet121	Densely Connected Network 121
DenseNet201	Densely Connected Network 201
GAN	Generative Adversarial Network
InceptionResNetV2	Inception + ResNet Variant 2
InceptionV3	Inception Variant 3
MobileNetV2	Mobile Network Variant 2
ReLU	Rectified Linear Unit
ResNet50	Residual Network 50
ResNet50V2	Residual Network 50 Variant 2
ROC	Receiver Operating Characteristic
SVM	Support Vector Machine
VGG16	Visual Geometry Group 16
Xception	Extreme Inception
